# Cavitated Conglomerate Mass in Silicosis Indicating Associated Tuberculosis

**DOI:** 10.1155/2010/293730

**Published:** 2010-08-05

**Authors:** Pedro Martins, Edson Marchiori, Gláucia Zanetti, Antonio Muccillo, Nina Ventura, Viviane Brandão, Mariana Leite Pereira, Carolina Pesce Lamas Constantino, Guilherme Abdalla, Romulo Varella de Oliveira, Rodrigo Canellas

**Affiliations:** Department of Radiology, Faculty of Medicine, Rio de Janeiro Federal University, Rua Thomaz Cameron, 438 Valparaiso, 25685.120 Petrópolis, RJ, Brazil

## Abstract

Silicosis is the most common occupational lung disease worldwide. It leads to respiratory impairment and may have associated infections that decrease pulmonary function. We describe the case of a 55-year-old man with chronic silicosis who presented with hemoptysis and a cavitated conglomerate mass. The final diagnosis was silicotuberculosis.

## 1. Introduction

Pneumoconiosis is caused by the accumulation of inhaled particulates that cause a reaction in the lung tissue. Silicosis, coal worker pneumoconiosis, and asbestosis are the three most common types of pneumoconiosis. Silicosis is a diffuse interstitial lung disease caused by inhalation of crystalline silica and is the most common occupational disease involving the lungs. The principal sources of industrial exposure to free silica are mining, quarrying, and tunneling; stonecutting, polishing, and cleaning monumental masonry; sandblasting and glass manufacturing; foundry work in pottery and porcelain manufacturing, brick lining, boiler scaling, and vitreous enameling. Diagnosis is based on a history of exposure to silica accompanied by a clinical and radiological profile consistent with the disease.

The disease is classified as chronic, accelerated, or acute, depending on the intensity and duration of the exposure to silica dust. The acute form, also known as silicoproteinosis, is caused by substantial short exposure to silica dust and usually manifests within 3 years after initial exposure. The predominant HRCT finding consists of bilateral air-space consolidation, often associated with ground-glass opacities and multiple small nodules. In the accelerated form, symptoms appear after 2 to 10 years, with radiological and pathological manifestations are identical to those of classic silicosis, despite the early onset and rapid progression. The chronic form manifests more than 10–20 years after exposure and is typically oligosymptomatic. However, it can evolve to progressive dyspnea on exertion [1–5]. In this case report, we describe a male patient with chronic silicosis and tuberculosis who was admitted to the hospital with hemoptysis.

## 2. Case Presentation

A 55-year-old man, who was a former smoker and had chronic silicosis caused by 14 years of work in the granite industry, was admitted to the hospital after an episode of hemoptysis. The amount of blood expectorated was estimated at more than 200 mL. He also had a one-month history of productive cough with blood in the sputum. He had previous history of tuberculosis, with the last episode approximately two years prior, when he received isoniazid, rifampin, and pyrazinamide for two months, followed by four months of isoniazid and rifampin. He had been asymptomatic since then.

On physical examination, the patient was emaciated and pale. His blood pressure was 100/70 mmHg, his pulse was 104 beats/min, and his respiratory rate was 20 breaths/min. Lung auscultation revealed rhonchi and wheezing in both lungs. No other alterations were seen in the physical examination. A blood chemistry screening was normal, including negative finding by anti-HIV. Immunodiffusion tests were negative for *Aspergillus*.

**Figure 1:**
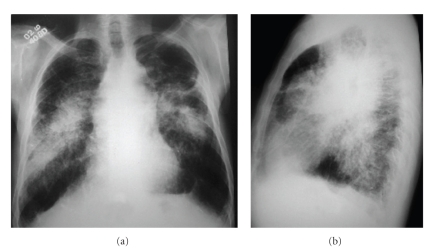
Chest radiographs in anteroposterior (a) and lateral (b) incidences showing bilateral perihilar conglomerate masses associated with multiple small nodules, predominantly in the upper and middle zones.

**Figure 2:**
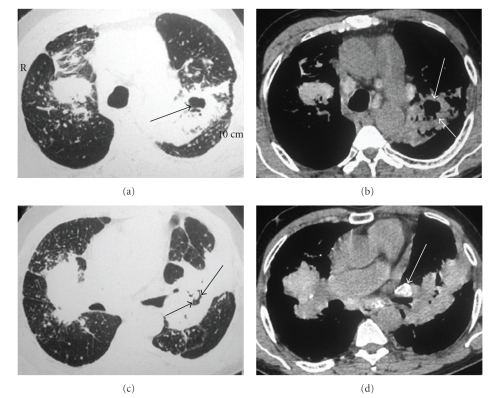
HRCT scan with pulmonary windows ((a) and (c)) showing conglomerate mass (progressive massive fibrosis) with adjacent small nodules in both lungs. Note that the left conglomerate mass presented with a cavitation (arrows). Areas of emphysema and an enlargement of the pulmonary artery trunk are seen. Mediastinal windows ((b) and (d)) also show calcifications in the hilar and mediastinal lymph nodes. Foci of calcification are present in the conglomerate masses.

A chest radiograph (Figure 1) and high-resolution computed tomography (HRCT) of the thorax (Figure 2) showed bilateral conglomerate masses, one with cavitation, associated with multiple small nodules, in addition to calcifications of the hilar and mediastinal lymph nodes, paracicatricial emphysema, architectural distortion, and enlargement of the central pulmonary arteries. No other parenchymal findings were suggestive of active tuberculosis.

The expectorated sputum was negative for acid-fast bacilli and the patient underwent a bronchoscopy that showed evidence of active bleeding. Direct examination of the bronchoalveolar lavage was negative for tuberculosis, fungi, or malignancy. Culturing was positive for *Mycobacterium tuberculosis* and negative for fungi.

Because of the enlarged pulmonary arteries, Doppler ecocardiography was performed, showing left ventricular diastolic function, mild tricuspid regurgitation, and pulmonary arterial hypertension with a systolic pulmonary artery pressure of 48 mmHg. The patient underwent an angiogram of the common bronchial trunk that showed marked hypervascularity and enlargement of left bronchial artery, which was selectively catheterized and subsequently embolized with polyvinyl alcohol particles (PVA). Bleeding stopped after embolization. The patient was treated for tuberculosis with isoniazid, rifampin, pyrazinamide, and ethambutol for two months, followed by seven months of isoniazid and rifampin. After the embolization and clinical treatment for tuberculosis, the patient progressed well, with improvement in his respiratory symptoms, and no rebleeding after a follow-up of two years.

## 3. Discussion

Chronic silicosis presents in two forms: simple and complicated. The simple form is characterized by multiple nodular opacities that are well defned and uniform in shape and attenuation, and range from 1 to 10 mm in diameter. They are distributed diffusely throughout both lungs, but tend to be most numerous in the upper lobe and posterior portion of the lung. The case presented here was the complicated form, which develops through the expansion and con*ﬂ*uence of individual silicotic nodules, and is characterized by the appearance of large opacities over one cm in diameter (conglomerate masses, or progressive massive fbrosis) [1–3, 5]. Hilar and mediastinal lymph node calcifcations are occasionally seen [2], as in our case. In some patients with the chronic form, disease progression can be rapid, evolving to death within a few months or years [3]. Patients with silicosis may develop impaired lung function [5].

The presence of conglomerate masses is associated with abnormal pulmonary function [5, 6]. In silicosis, lung function abnormalities correlate better with emphysematous changes than with nodular changes [7–10]. In nonsmokers, obstructive changes occur only in the presence of advanced silicosis [8, 11]. Areas of emphysema cause an increased pulmonary vascular resistance due to alveolar hypoxia leading to pulmonary hypertension [12]. Although the patient in this case did not undergo right heart catheterization, which is the diagnostic standard for measuring pulmonary hemodynamic parameters, the hypothesis of pulmonary hypertension was supported by the high systolic pulmonary artery pressure estimated by Doppler ecocardiography, and pulmonary artery enlargement shown by HRCT.

The risk of developing pulmonary tuberculosis is reported to be from 2.8 to 39 times higher for patients with silicosis than for healthy controls [3, 10, 13–17]. In silicosis patients, excluding the coexistence of active tuberculosis is extremely important, because this would indicate a treatment other than chemoprophylaxis [3]. Establishing a diagnosis of tuberculosis in these cases can be difficult, and the presence of systemic symptoms should raise the suspicion of associated infection [18]. We suspected silicotuberculosis in our case, because the patient had a previous history of tuberculosis associated with a one-month history of productive cough and hemoptysis, and the computed tomography showed cavitation of the conglomerate mass. In a series of 44 patients [18] with silicosis and conglomerate masses who underwent chest HRCT, cavitations were noted in 8 patients, and of these, 6 had concomitant tuberculosis. Occasionally, cavitation due to ischemic necrosis may occur in a conglomerate mass [1, 2].

Tuberculosis, including tuberculosis bronchiectasis, bronchogenic carcinoma, and chronic inflammatory lung disease due to bronchiectasis, cystic fibrosis, or aspergillosis are the most common causes of massive hemoptysis [19]. Exclusion of a fungus ball or aspergilloma is important, because hemoptysis is the most clinically important consequence of aspergiloma [20]. Aspergilloma was excluded in this case, and the patient underwent a selective angiogram demonstrating a left hypertrophied bronchial artery, which was embolizated with PVA, the material most frequently used worldwide for bronchial artery embolization [19].

Severe hemoptysis is a common presentation in acute and chronic cavitary pulmonary tuberculosis. Disease activity does not appear to affect the frequency or severity of hemoptysis [21]. Many severe hemoptysis patients are poor surgical candidates, because of poor respiratory reserves from extensive disease, as in our case, and systemic arterial embolization is a recognized mode of management for most of these patients [22]. The source of massive hemoptysis is bronchial circulation in 90% of cases [19]. The hemoptysis occurs because pulmonary circulation is reduced or occluded at the level of the pulmonary arterioles, near an area of active or chronic in*ﬂ*ammation. The bronchial arteries proliferate and enlarge to replace the pulmonary circulation, which may rupture, causing extravasation into the respiratory tree, resulting in massive hemoptysis [19]. Recurrent bleeding is common in patients with chronic tuberculosis. In one study of 103 patients who underwent bronchial arterial embolization, 16 (15.5%) required repeat embolization, and all had hemoptysis due to chronic tuberculosis [23].

## 4. Conclusion

Despite the priority of International Labour Organization to eliminate silicosis, the prevalence of this disease is still high in developing countries, as is the prevalence of tuberculosis. Excluding associated infections is important in silicosis patients with new symptoms, since these associations are common and change the case management. Chest X-ray and computed tomography associated with sputum smear microscopy should be the first diagnosis methods performed for diagnosing active tuberculosis. The presence of cavitation in a conglomerate mass is an important indication of associated tuberculosis.
